# Long noncoding RNA HCG18 inhibits the differentiation of human bone marrow-derived mesenchymal stem cells in osteoporosis by targeting miR-30a-5p/NOTCH1 axis

**DOI:** 10.1186/s10020-020-00219-6

**Published:** 2020-11-11

**Authors:** Mingxue Che, Weiquan Gong, Yao Zhao, Mingxi Liu

**Affiliations:** 1 Department of Spine Surgery, The First Hospital of Jilin University, No.1 Xinmin Street, Changchun, 130021 Jilin Province China; 2 Department of Joint Surgery, The First Hospital of Jilin University, No.1 Xinmin Street, Changchun, 130021 Jilin Province China; 3 Department of Orthopedic Traumatology, The First Hospital of Jilin University, No.1 Xinmin Street, Changchun, 130021 Jilin Province China

**Keywords:** Osteoporosis, Human BMSCs, HCG18, miR-30a-5p, NOTCH1

## Abstract

**Background:**

Recent studies have demonstrated that long non-coding RNAs (LncRNAs) can influence bone cell differentiation and formation. However, it is unclear whether lncRNA HCG18 is involved in osteoporosis (OP). This study was conducted to investigate the regulation of HCG18 in osteogenic differentiation of bone marrow mesenchymal stem cells (BMSCs).

**Methods:**

BMSCs were isolated and cultured from mouse pathological models and osteoporosis patients. RT-qPCR was performed to detect the expression of HCG18 and miR-30a-5p in BMSCs. The interaction between HCG18 and miR-30a-5p was analyzed by dual luciferase assay and RNA pulldown assay. The interaction between miR-30a-5p and NOTCH1 3′-UTR was analyzed by dual luciferase assay. RT-qPCR and Western blotting were used to detect the expression of osteogenic genes Runx2, OCN and OPN. Hindlimb-unloaded (HU) mice model was established, and HCG18 was knocked down on bone-formation surfaces by using lentivirus mediated shRNA transfection.

**Results:**

The expression of HCG18 was increased in BMSCs of OP patients, while the expression of miR-30a-5p was decreased. The expression of HCG18 and miR-30a-5p was negatively correlated in BMSCs. During the differentiation from BMSCs to osteoblasts, the expression of HCG18 was significantly downregulated, and the expression of miR-30a-5p was significantly upregulated. Overexpression of HCG18 was able to reverse the osteogenic-induced upregulation of miR-30a-5p expression, and knockdown of HCG18 further promoted the expression of miR-30a-5p. In addition, miR-30a-5p partially abolished the effect of HCG18 on osteogenic differentiation of BMSCs. NOTCH1 was a target protein of miR-30a-5p, and upregulation of NOTCH1 reversed the effect of miR-30a-5p on osteogenic differentiation of BMSCs. Furthermore, this study found that lentivirus mediated HCG18 knockdown on the bone-formation surfaces of hindlimb-unloaded (HU) mice partially alleviated unloading-induced bone loss

**Conclusions:**

HCG18 inhibited osteogenic differentiation of BMSCs induced by OP via the miR-30a-5p/NOTCH1 axis. HCG18 can be identified as a regulator of osteogenic differentiation of BMSCs.

## Background

Osteoporosis (OP) is a metabolic bone disease characterized by decreased bone mass and degeneration of bone tissue, leading to increased bone fragility and easy fracture (Melton et al. 1997; Gielen et al. 2017). It is more common in postmenopausal women and the elderly. Serious complications of OP occur in fragile fractures (Compston et al. 2017). Research data have shown that the risk of recurrent vertebral fractures after a hip fracture will increase by 2.5 times. Older people have a higher risk of fracture because of poor bone quality, calcium and vitamin D deficiency (Leder 2017; Qaseem et al. 2017). OP also causes social, family and economic burdens, so early prevention and treatment of OP is particularly important.

The occurrence of OP is closely related to the biological behavior of bone marrow mesenchymal stem cells (BMSCs), such as decreased number of osteoblasts and the ability of differentiation, the increasing of apoptosis or the enhancement of adipogenic differentiation ability (Hang and Xia 2014; Liu et al. 2014). In a clinical study, peripheral blood mesenchymal stem cells (MSCs) from OP patients and healthy individuals were cultured in vitro for 15 days. It was found that peripheral blood MSCs in osteoporosis patients increased compared with that in healthy controls, and the expression of osteogenic differentiation factor Runx2 was downregulated (Deng et al. 2010). In the glucocorticoid-induced OP mouse model, the osteogenic differentiation of BMSCs is found to be weakened. Therefore, regardless of the factors caused by osteoporosis, improving the osteogenic differentiation of BMSCs can help delay the development of osteoporosis (Jing et al. 2018; Liu et al. 2018). At present, most drugs for the treatment of OP are bone resorption inhibitors. Only small doses of parathyroid hormone (PTH) can promote bone formation. Because of the high price, inconvenient injection and poor efficacy, it has not been widely used (Huang et al. 2010). It is important to develop new treatments and preventive measures.

Long non-coding RNAs (LncRNAs) play critical roles in biological processes and regulation of gene expression, such as transcriptional regulation, epigenetic modification and post-translational modification (He et al. 2015; Guan et al. 2017). Dysregulation of lncRNAs is closely related to OP (Mei et al. 2019). As a biomarker in OP bone metabolism, the mechanism of lncRNA regulation of bone metabolism-related signaling pathways is complex (Wang et al. 2017). With the development of epigenetics studies, characterization of lncRNAs will provide a new target for the diagnosis, treatment and prognosis of OP. Studies have found a significant correlation between osteogenic differentiation of lncRNA and BMSCs (Wang et al. 2018; Jin et al. 2017). LncRNA HCG18 (HCG18) is a newly discovered lncRNA, which has been found to be abnormally expressed in a variety of malignant diseases (Xi et al. 2017). However, the mechanism by which HCG18 regulates osteogenic differentiation of OP BMSCs is unclear.

Micro RNA (miRNA) have been proved to play a part in a variety of cell biological processes, such as cell differentiation and metabolism (Smirnova et al. 2015; Dweep et al. 2011). MicroRNAs have a central role in the metabolic activities of bone cells in organisms (Mahmood et al. 2011). MicroRNAs maintain the metabolic balance of bone by regulating the phenotypic differentiation of mesenchymal stem cells and hematopoietic stem cells, ultimately affecting bone metabolic homeostasis and bone formation (Laxman et al. 2015). MiR-30a-5p is a newly discovered miRNA molecule that is closely related to central nervous system function, which has a regulatory role in hippocampal neuronal apoptosis and neuroprotection (Croce et al. 2013). However, little is known about whether osteogenic defects in BMSCs are associated with abnormal expression of miR-30a-5p. Recent studies have found that lncRNAs may exert their roles in gene regulations through miRNA adsorption processes (Paraskevopoulou and Hatzigeorgiou 2016). The Notch signaling pathway is a highly conserved signaling pathway in cell differentiation potential, cellular self-renewal capacity and apoptosis (Nowell and Radtke 2017). Notch1 is one of the receptors of Notch, and studies have shown that inhibition of Notch1 reduces osteoblast proliferation and differentiation (Ann et al. 2011; Zanotti and Canalis 2014). Furthermore, Notch1 has been reported to act as a target gene of miR-30-5p in ovarian cancer and macrophages (Wang et al. 2019; Miranda et al. 2018). Also, we performed Bioinformatics analysis on starbase v2.0 (https://starbase.sysu.edu.cn/) to screen targets of HCG18. It showed that seven miRNAs, including miR-140-3p, miR-214-5p, miR-133a-3p, miR-324-5p, miR-145-5p, miR-142-5p and miR-30a-5p, are related with osteogenesis and osteoporosis from a bioinformatics website (https://www.genecards.org/). We initially performed a pre-experiment to investigate the effect of HCG18 overexpression on these miRNAs. The results suggested that overexpression of HCG18 down-regulated the expression of miR-30a-5p in HEK293T cells, while had no influence on other miRNAs. Therefore, we hypothesized that HCG18 might cause abnormal osteogenic differentiation of BMSCs through the miR-30a-5p/Notch1 axis, inhibiting its mediated bone formation and causing osteoporosis. The main purpose of this study was to investigate the mechanism of HCG18 in osteogenic differentiation of mesenchymal stem cells, and to verify the role and relationship of miR-30a-5p/Notch1 axis in osteogenic differentiation of HCG18-mediated BMSCs.

## Results

### The expression of HCG18 and miR-30a-5p in hindlimb unloaded (HU) mice and OP patients

**Fig. 1:**
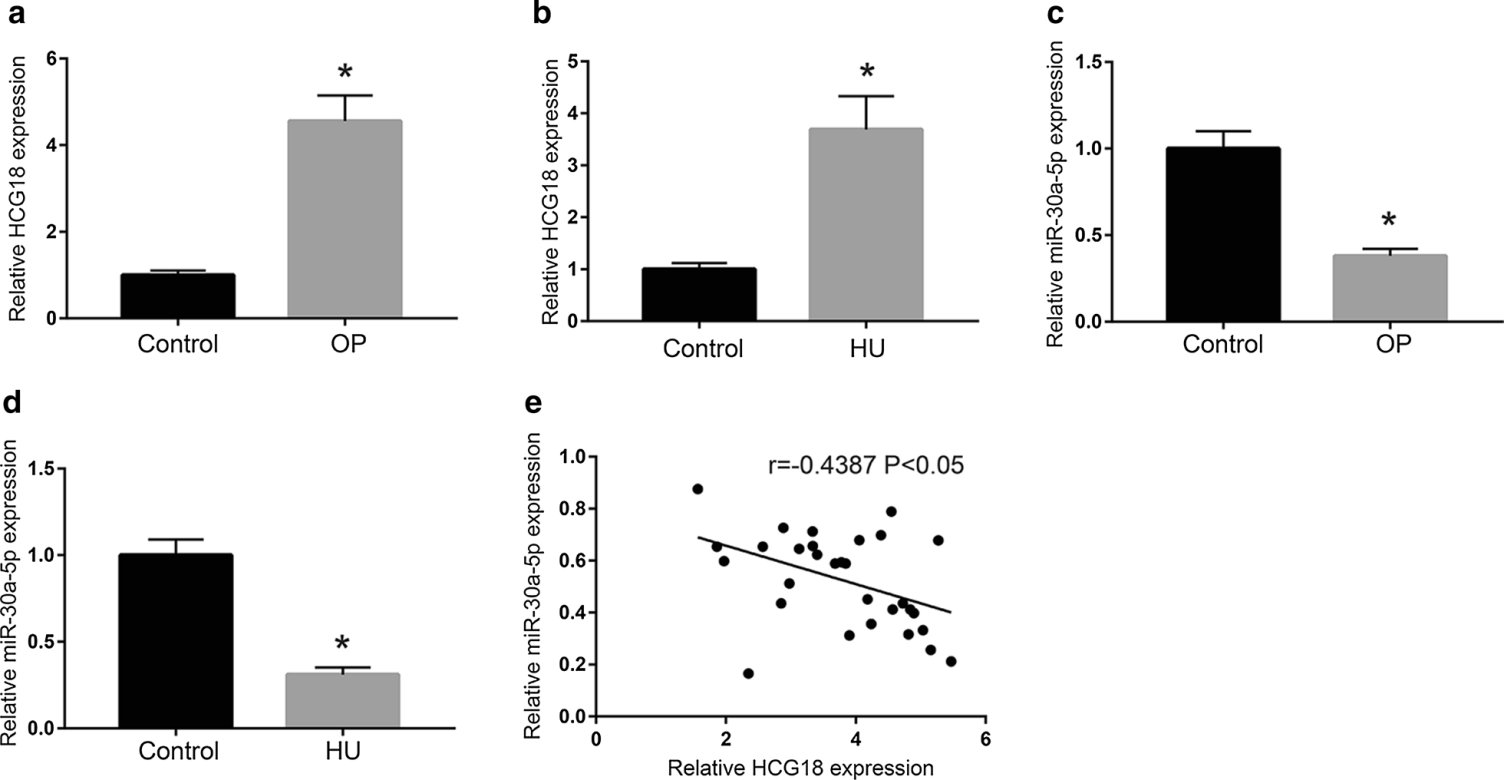
Expression of HCG and miR-30a-5p in BMSCs of OP and HU. **a**, **b** Expression of HCG18 in OP (n = 30) and HU (n = 30). Control: n = 30. **c**, **d** Expression of miR-30a-5p in HU (n = 30) and OP (n = 30). Control: n = 30. **e** The expression of HCG18 in BMSCs was negatively correlated with the expression of miR-30a-5p (n = 30). *P < 0.05 vs. the control group

The expression of HCG18 and miR-30a-5p in BMSCs was detected by qRT-PCR. As shown in Fig. 1a, b, the expression of HCG18 in BMSCs of HU and OP groups was significantly increased compared with control group (*P* < 0.05). As shown in Fig. 1c, d, the expression of miR-30a-5p in BMSCs of HU and OP was significantly reduced (*P* < 0.05). In addition, the expression of miR-30a-5p was negatively correlated with HCG18 in BMSCs of PMOP (n = 30) (Fig. 1e).

### The relationship between the expression of HCG18/miR-30a-5p and the osteogenic induction time of BMSCs

**Fig. 2:**
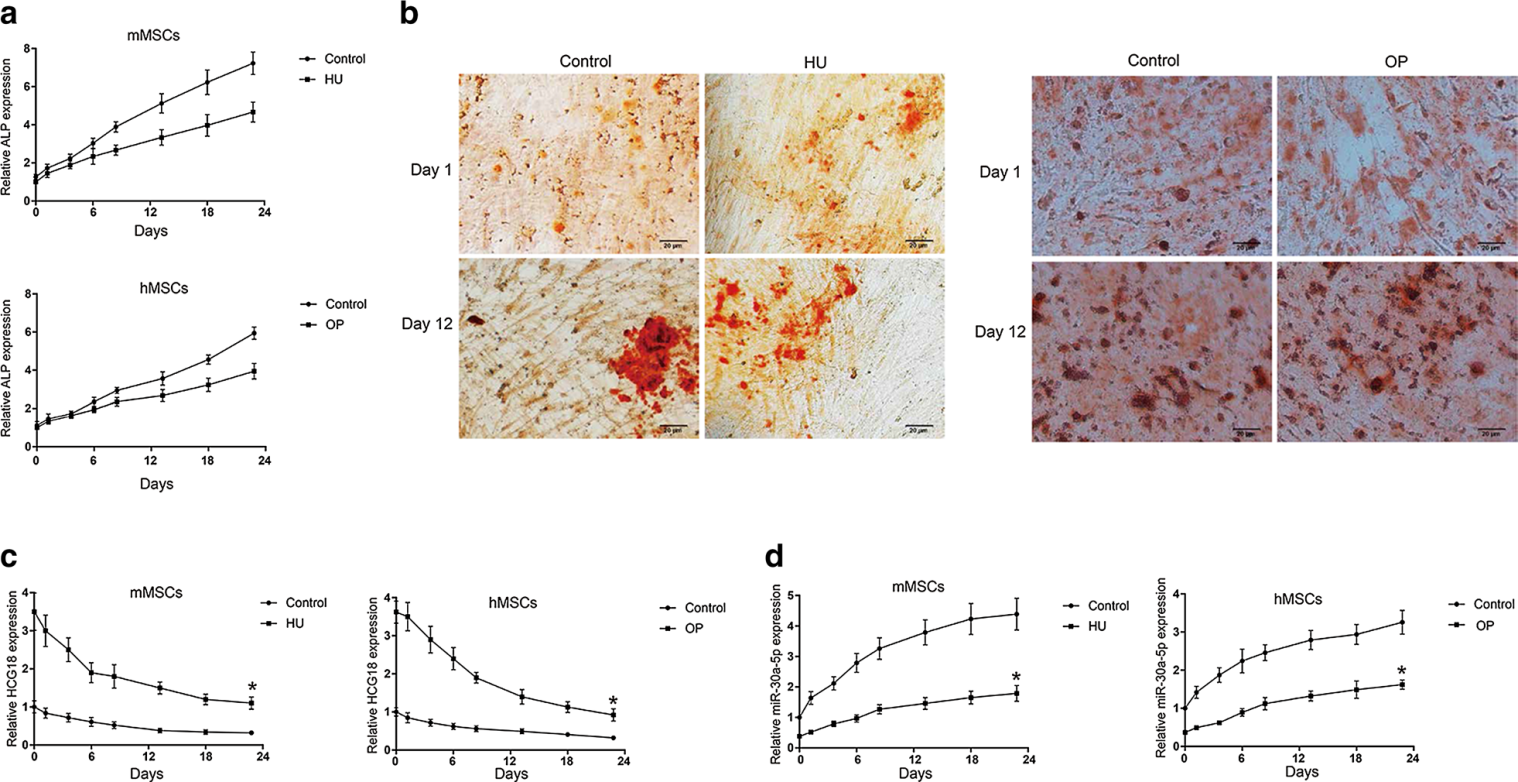
Expression of HCG18 and miR-30a-5p in BMSCs of HU mice and OP patients. Expression of **a** ALP, **c** HCG18 and **d** miR-30a-5p were detected in BMSCs during 23 days’ induction. **b** Presentative figures of ALP staining. ALP staining was performed at days 1 and 12, respectively (magnification, ×100). White arrows indicate areas of ALP activity. Bars represent standard deviation from 6 independent biological replicas of the experiment

The expression of ALP, HCG18 and miR-30a-5p in the cells were detected by qRT-PCR. As shown in Fig. 2a, after 23 days’ induction, the expression of ALP in mMSCs group and hMSCs group gradually increased in a time-dependent manner. Furthermore, the expression of ALP in OP or HU-BMSCs was significantly decreased than that in the control group (Fig. 2a). ALP staining results shown in Fig. 2b were consistent with the expression of ALP. As shown in Fig. 2c, d, after 23 days’ induction, the expression of HCG18 was gradually down-regulated in the mMSCs group and the hMSCs group, while the expression of miR-30a-5p was gradually increased in a time-dependent manner. Furthermore, the expression of HCG18 was significantly up-regulated in HU and OP-BMSCs groups compared with control group, while miR-30a-5p was down-regulated.

### Effects of HCG18 and miR-30a-5p on osteogenic differentiation of BMSCs

**Fig. 3:**
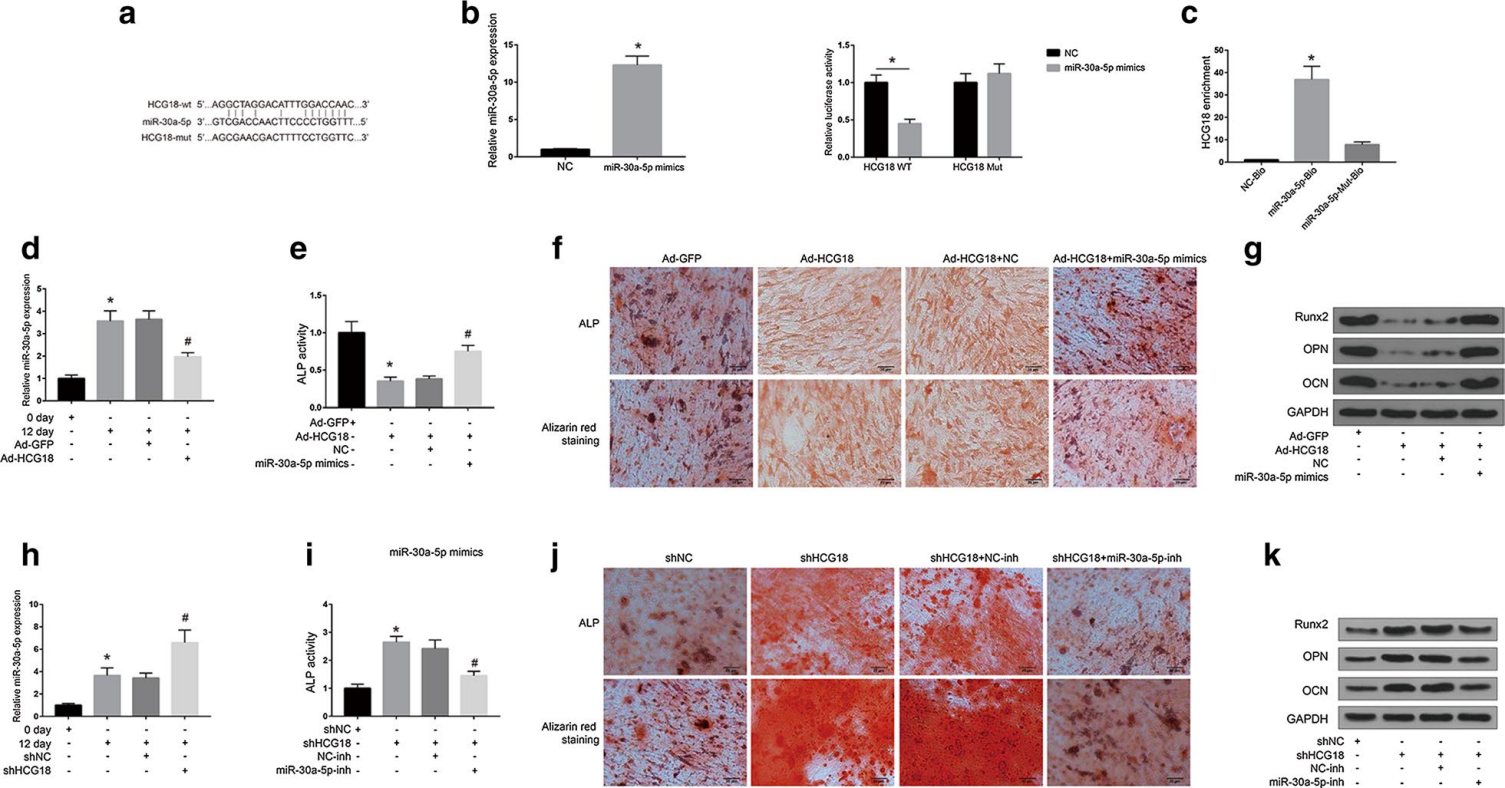
Effect of HCG18 and miR-30a-5p on osteogenic differentiation of BMSCs. **a** Putative binding sites for HCG18 and miR-30a-5p. **b** Left panel, expression of recombinant adenovirus transfected with HCG18. Right panel, luciferase activity of HCG18-wt and HCG18-mut in HEK-293T cells treated with miR-665 mimic or NC. **c** RNA pull-down assay indicated that HCG18 could be enriched by biotin-labelled miR-30a-5p but not biotin-labelled miR-30a-5p mutant type. **d** HCG18 overexpression inhibited miR-30a-5p expression in OP-BMSCs. **e** ALP activity assay and **f** ALP staining and Alizarin Red S staining were performed on OP-BMSCs to investigated the effects of HCG18 and miR-30a-5p co-transfection on the osteogenic differentiation of OP-BMSCs. **g** Determination of the expression of specific markers for osteogenic differentiation (Runx2, OCN, OPN) of OP-BMSCs co-transfected with HCG18 and miR-30a-5p. **h** Knockdown of HCG18 promoted miR-30a-5p expression in OP-BMSCs. **i** ALP activity assay and **j** ALP staining and Alizarin Red S staining were performed on OP-BMSCs to investigate the effects of shHCG18 and miR-30a-5p-inhibitor co-transfection on the osteogenic differentiation of OP-BMSCs. **k** Determination of the expression of specific markers for osteogenic differentiation (Runx2, OCN, OPN) of OP-BMSCs co-transfected with shHCG18 and miR-30a-5p-inhibitor. * *P* < 0.05. The OP-BMSCs were from one human subject and the experiments were performed in three replicates

Next, the interaction of HCG18 and miR-30a-5p in OP-BMSC cells was assessed. The online program starbase v2.0 (https://starbase.sysu.edu.cn/) was used, and miR-30a-5p was identified as a potential target for HCG18 (Fig. 3a). As shown in Fig. 3b, the expression of miR-30a-5p was significantly increased in the miR-30a-5p mimics group compared with NC group (*P* < 0.05). Moreover, overexpression of miR-30a-5p significantly inhibited the luciferase activity of HCG18-WT, while the mutation of the matching site in the 3′-UTR of HCG18 had no significant effect on luciferase activity, indicating that the interaction between the binding sites of miR-30a-5p and HCG18 3′-UTR can directly regulate the expression of the luciferase reporter gene. In addition, RNA pull-down assay showed that miR-30a-5p-Bio was able to significantly enrich HCG18 (*P* < 0.05), while miR-30a-5p-Mut-Bio could not enrich HCG18 (Fig. 3c). In addition, as shown in Fig. 3d, the expression of miR-30a-5p was significantly increased when the osteogenic differentiation induction medium was continued for 12 days (*P* < 0.05), while Ad-HCG18 significantly down-regulated the expression of miR-30a-5p in OP-BMSCs (*P* < 0.05). As shown in Fig. 3e–g, ALP activity, ALP staining, Alizarin red staining and osteogenic-related gene (ALP, OCN and OPN) protein expression were significantly decreased in the Ad-HCG18 group compared with control group (*P* < 0.05). While ALP activity, ALP staining, Alizarin red staining and osteogenic related gene (ALP, OCN and OPN) protein expression were significantly increased in the miR-30a-5p mimics group (*P* < 0.05). Furthermore, HCG18 was knocked down in OP-BMSCs, and as shown in Fig. 3h, shHCG18 significantly up-regulated the expression of miR-30a-5p (*P* < 0.05). ALP activity, ALP staining, Alizarin red staining and osteogenic-related gene (ALP, OCN and OPN) protein expression was significantly increased in the sh-HCG18 group compared with control group (*P* < 0.05), while this elevation was reversed by knockdown of miR-30a-5p (Fig. 3i–k).

### Effects of HCG18 and miR-30a-5p on osteogenic differentiation of BMSCs were mediated by NOTCH1

**Fig. 4:**
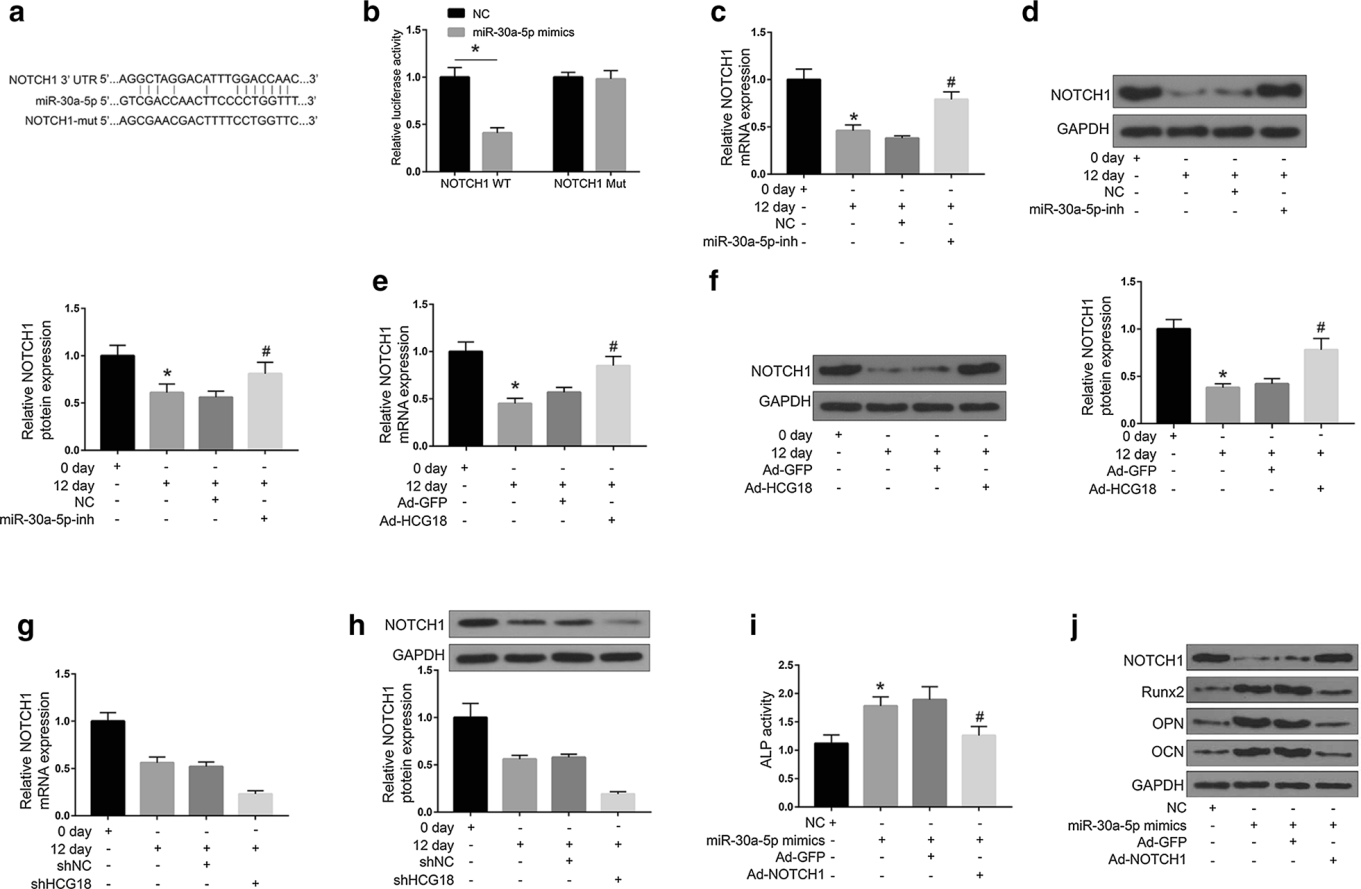
Effect of miR-30a-5p and NOTCH1 on osteogenic differentiation of BMSCs. **a** Putative binding sites for NOTCH1 and miR-30a-5p. **b** Luciferase activity of NOTCH1-wt and NOTCH1-mut in miR-30a-5p mimics or NC-treated BMSC cells. **c** Effect of overexpression of miR-30a-5p on the expression of NOTCH1. **d** Effect of overexpression of miR-30a-5p on the expression of NOTCH1 protein. **e** Effect of overexpression of HCG18 on the expression of NOTCH1. **f** Effect of overexpression of HCG18 on the expression of NOTCH1. **g** Effect of HCG18 knockdown on NOTCH1 mRNA expression. **h** Effect of HCG18 knockdown on NOTCH1 protein expression. **i** ALP activity assay. **j** Determination of the expression of osteogenic differentiation specific markers (Runx2, OCN, OPN). * *P* < 0.05. The OP-BMSCs were from one human subject and the experiments were performed in three replicates

We predicted by online program starbase v2.0 (https://starbase.sysu.edu.cn/), and NOTCH1 was identified as a potential target for miR-30a-5p (Fig. 4a). To analyze whether miR-30a-5p directly regulated NOTCH1, a luciferase reporter construct of NOTCH1 (NOTCH1-wt) and mutant form (NOTCH1-mut) were generated (Fig. 4b). The results showed that overexpression of miR-30a-5p significantly inhibited the luciferase activity of NOTCH1-WT, but not its mutant form. These results indicated that miR-30a-5p and NOTCH1 could directly interact with each other. Next, we investigated the effects of HCG18/miR-30a-5p in OP-BMSCs. As shown in Fig. 4c, d, when OP-BMSCs were cultured in the osteogenic differentiation induction medium for 12 days, the mRNA and proteinexpression of NOTCH1 were significantly decreased (*P* < 0.05). While the mRNA of NOTCH1 and protein expression were significantly increased in the miR-30a-5p-inh group (*P* < 0.05). Furthermore, as shown in Fig. 4e, f, overexpression of HCG18 could significantly up-regulate the expression of NOTCH1 at both mRNA and protein levels (*P* < 0.05). In contrast, knockdown of HCG18 significantly downregulated the expression of NOTCH1 at both mRNA and protein levels (*P* < 0.05, Fig. 4g, h).

The interaction of miR-30a-5p and NOTCH1 in osteogenic differentiation of BMSCs was further analyzed. As shown in Fig. 4i, j, compared with the control group, ALP activity and Runx2, OCN and OPN protein expression were significantly increased in the miR-30a-5p mimics group (*P* < 0.05). The ALP activity and Runx2, OCN and OPN protein expression in the Ad-NOTCH1 group were significantly reduced (*P* < 0.05).

### HCG18 knockdown improves bone phenotypes in HLU mice

**Fig. 5:**
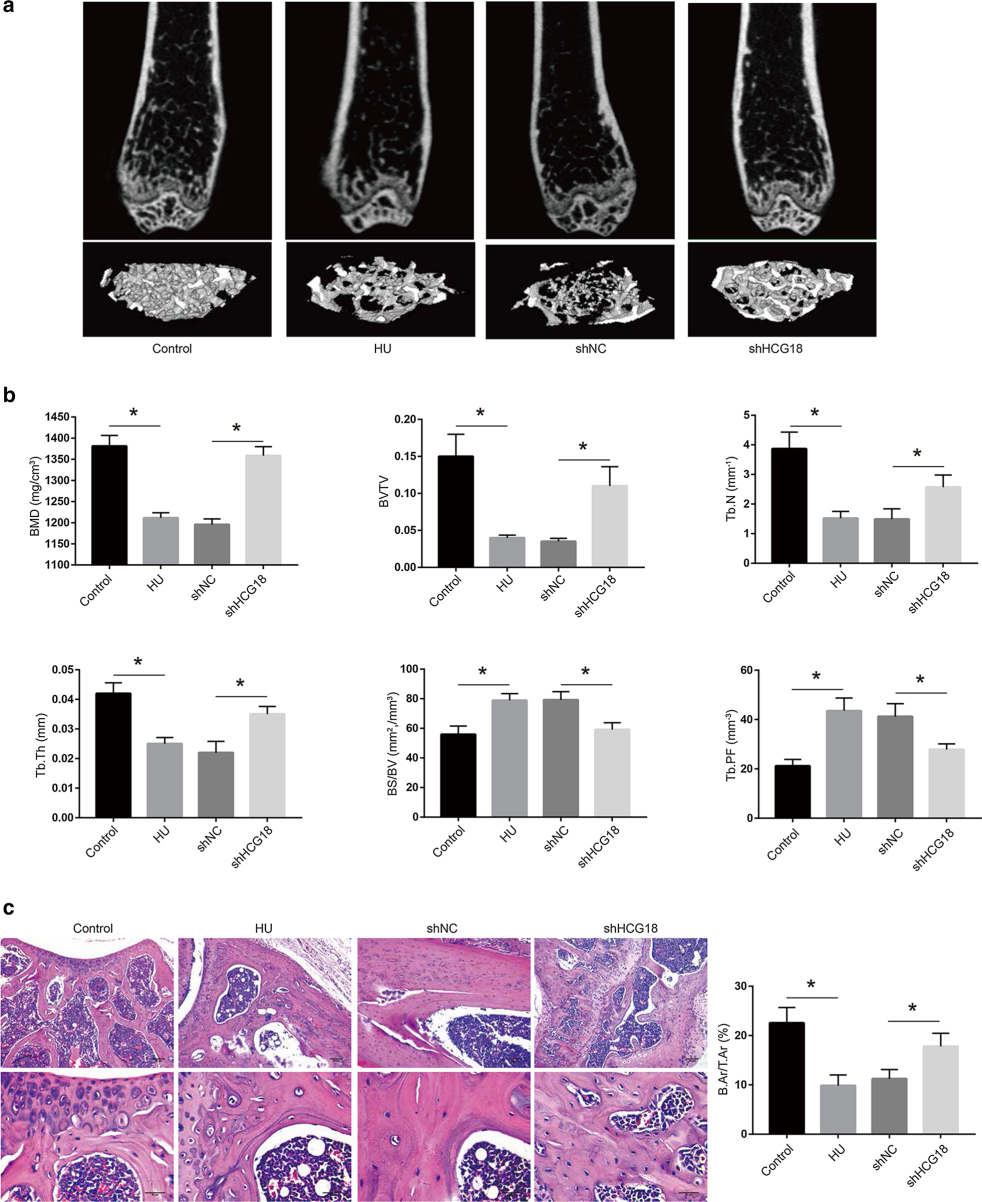
Bone-targeted shHCG18 improves trabecular bone phenotypes in HU mice. **a** Representative images of micro-CT 2D sections and 3D reconstruction of distal femurs of mice in the indicated groups (n = 5). **b** Micro-CT measurement of bone mineral density (BMD), the ratio of bone volume to total volume (BV/TV), the ratio of bone surface to bone volume (BS/BV), trabecular bone number (Tb.N), trabecular thickness (Tb.Th) and trabecular bone pattern factor (Tb.PF) (n = 5). **c** H&E staining showing trabecular microarchitecture and quantitative analysis of the bone area/total area on the proximal side of the growth plate in the distal femurs of mice in the indicated groups (n = 5). Scale bars: 300 tain. **d** Representative load deflection curves for the respective groups (n = 5). **e** The maximum load, stiffness and elastic modulus were calculated (n = 5). All data are the mean ± SD. *P < 0.05

Next, we further explored the effects of bone-targeted HCG18 knockdown on bone phenotypes. Micro-CT assays revealed that the significant decreases in bone mineral density (BMD), the ratio of bone volume to total volume (BV/TV), trabecular bone number (Tb.N), trabecular thickness (Tb.Th) and the increases in the ratio of bone surface to bone volume (BS/BV) and trabecular bone pattern factor (Tb.PF) induced by HLU were efficiently attenuated in the HCG18 knockdown group (Fig. 5a, b). H&E staining of the proximal side of the growth plate in the distal femurs further confirmed that HCG18 knockdown counteracted the low-bone-mass phenotype of HLU mice, as quantified by the ratio of bone area to total area (B.Ar/T. Ar) (Fig. 5c). These data demonstrated that the low bone mass and deterioration of the trabecular microarchitecture in HLU mice were alleviated by bone-targeted HCG18 knockdown.

## Discussion

Aging is the process of physical deterioration of the body and can be accompanied by various diseases, including osteoporosis, arthritis, cardiovascular atherosclerosis, Alzheimer’s disease and malignant tumors. With the increasing aging population worldwide, common diseases related to old age, such as osteoporosis, have been causing widespread concern. As a systemic metabolic disease, OP causes microarchitectural deterioration of bone tissue, leading to increased bone fragility and risk of bone fractures (He et al. 2017). Different factors contribute to the onset of osteoporosis, including genetic, nutritional and lifestyle factors among others (Lv et al. 2018).

HMSC have the potential of self-renewal and multi-directional differentiation and belong to multipotent stem cells, which are mainly isolated from bone marrow (Miao et al. 2013; Morigi et al. 2010). Under certain induction conditions, BMSCs can differentiate into a variety of mature cells, such as osteoblasts, nerve cells and hepatocytes (Zhu et al. 2012). The differentiation of hMSC (especially into osteoblasts) is strictly regulated by mechanical and molecular signaling pathways and has great potential in the field of stem cell therapy for osteogenesis disorders (Tansriratanawong et al. 2014). However, the key regulatory sites and regulatory mechanisms of osteogenic differentiation of BMSC need further study.

As the number of OP patients is growing year by year, there is a constant need for innovation in diagnosis and treatment. Studies on lncRNAs and OP have been reported (Mei et al. 2019). LncRNAs AK028326 and AK141205 were found to induce multifunctional differentiation of mouse embryonic stem cells (mESCs) (Sheik et al. 2010). LncRNA MALATl can bind to the promoters of Spl and LTBP3 to promote the expression of LTBP3 and maintain myeloma-derived mesenchymal stem cell activity (Landi et al. 2014). HCG18 is a recently studied lncRNA, which is abnormally expressed in many diseases (Si-Yu et al. 2019). In this study, it was found that the expression of HCG18 in BMSCs was upregulated in HU and OP. After 23 days’ induction, the expression of HCG18 were gradually decreased in the mMSCs group and the hMSCs group. It was suggested that HCG18 might be involved in the regulation of BMSCs to differentiate into osteoblasts.

Studies have found that the interactions between lncRNAs and miRNAs play a part in the occurrence of diseases (Xiao et al. 2018). There is growing evidence showed that miRNAs bind to the 3′-terminus of key transcription factors associated with osteoblast differentiation. It plays a critical role in directional differentiation of mesenchymal stem cells into osteoblasts and bone formation and development by inhibiting the translation and expression of target RNA or degrading target RNA (Hu et al. 2015). For example, the expression of miR-23a-5p was upregulated in the differentiation of BMSCs into osteoblasts. It regulates the downstream mitogen-activated protein kinase-13, thus promoting differentiation of osteoblasts (Ren et al. 2018). MiR-30a-5p can regulate the differentiation of cells (Franzetti et al. 2013). In this study, it was found that the expression of HCG18 in BMSCs was significantly decreased in HU and OP. After 23 days’ induction, the expression of HCG18 were gradually increased in the mMSCs group and the hMSCs group. And HCG18 acted as a competing endogenous RNA (CeRNA) for miR-30a-5p. Moreover, overexpression of HCG18 inhibited osteogenic differentiation of BMSCs, which was characterized by decreased ALP activity and Runx2, OCN and OPN protein levels. However, knockdown of miR-30a-5p increased BMSCs.

The Notch1 signaling pathway is one of the important pathways that influences cell fate determination (Sanchez-Martin and Ferrando 2017). Neighboring cells can regulate the differentiation, proliferation and apoptosis of various cells through the Notch1 signaling pathway. Activation and blockade of Notch1 signaling pathway can affect the differentiation of BMSCs (Kang et al. 2017). It has been found that Notch1 signaling in hMSCs can promote the osteogenic differentiation of hBMSCs, but it also indirectly affects osteoclast proliferation and increases bone resorption (Fan et al. 2014). In this study, it was found that Notch1 might be a downstream target gene of miR-30a-5p. MiR-30a-5p inhibited the expression of NOTCH1. In addition, HCG18 promoted the expression of NOTCH1. Moreover, overexpression of miR-30a-5p can promote osteogenic differentiation of BMSC. However, overexpression of NOTCH1 inhibited osteogenic differentiation of BMSC.

## Conclusion

HCG18 inhibited osteogenic differentiation of OP induced by BMSCs via the miR-30a-5p/NOTCH1 axis. It lays a theoretical foundation for further elucidating the molecular and biochemical mechanism of BMSCs directed osteogenic differentiation in human bone marrow.

## Methods

### Isolation and culture of BMSCs

Bone marrow samples were obtained from discarded femoral head tissues during THA with or without OP (n = 30). All patients signed the informed consent. This study was approved by the Ethics Committee of the First Hospital of Jilin University's. Inclusion criteria were patients accepting total hip arthroplasty (THA) with or without osteoporosis. Exclusion criteria are cancer, rheumatoid arthritis and other metabolic diseases such as diabetes, hyperparathyroidism, hyperthyroidism and renal disease. We used the whole bone marrow adherence method to isolate hBMSCs. Clinical examination excluded other metabolic diseases. Human BMSCs were subcultured in standard growth medium containing phenol red-free alpha-MEM (HyClone, Logan, UT), 10% FBS-HI (Qifa, Shanghai, China), 100 U/mL penicillin (HyClone). The medium was changed every 2 days. In BMSC osteogenic induction experiments, 10% FBS-HI was reduced to 1% FBS-HI to avoid proliferation and differentiation. 50 μg/mL ascorbic acid (Sigma-Aldrich, St. Louis, Missouri), 10 mM beta glycerophosphate and 0.1 μg/mL dexamethasone (Sigma-Aldrich) were added. The detailed characteristics of the study subjects are summarized in Table 1.

**Table 1:** Characteristics of the study subjects

Traits	OP group (n = 30)	Control group (n = 30)	P value
Age (years)	61.2 ± 3.1	58.6 ± 2.5	0.13
Height (cm)	173.6 ± 2.5	170.6 ± 3.1	0.06
Weight (kg)	58.3 ± 4.9	65.9 ± 4.5	0.012*
Forearm T-score	− 1.13 ± 1.26	0.58 ± 1.12	0.024*
Hip T-score	− 3.12 ± 0.59	1.13 ± 1.59	< 0.001*
Femoral neck T-score	− 3.05 ± 0.79	0.35 ± 0.97	< 0.001*
Femur T-score	− 1.84 ± 0.79	0.79 ± 0.56	< 0.001*

^*^P < 0.05 by student’s test

### HU model

Male C57BL/6J mice (6-month old) were purchased from Shanghai Laboratory Animal Research Center (Shanghai, People’s Republic of China). Mice (n = 30) were housed under standard conditions (lighting/dark cycle of 12/12 h, control temperature 21 °C) for 1 week. The Morey-Holton method was used to suspend the tail of suspension group and the tail suspension administration group to the cage beam, so that the trunk was at an angle of 30° to the ground, the body could rotate 360° horizontally, and the forelimbs were free to move. After the tail was suspended for 3 weeks, the mice were anesthetized. All animal experiments were approved by the clinical committee of the First Hospital of Jilin University and conducted in accordance with the approved guidelines. BMSCs were isolated from bone marrow by whole bone marrow adhesion. The mice were sacrificed by cervical dislocation after the experiment. The culture conditions and osteogenic induction of mouse BMSCs were the same as that of culturing human BMSCs.

### RNA purification and qRT-PCR

Total RNAs in cells were extracted using TRIzol reagent (Boyao, Shanghai, China). After reverse transcription, qRT-PCR was performed using a ViiA™ 7 real-time PCR system (Life Technologies, Grand Island, NY). The expression of HCG18 and miR-30a-5p were calculated by the 2^−ΔΔCT^ method (Livak and Schmittgen 2000). The expression of LOXL1-AS1 were normalized to GADPH, while the expression of miR-515-5p were normalized to U6. qRT-PCR specific experimental methods were performed with reference to the literature (). The primers used in this study were as follows:

HCG18-F: 5′-TGAAGTCGACGAGAGGAGC-3′

HCG18-R: 5′-ACTAGTCGAGAGTGAGGTGC-3′

MiR-30a-5p-F: 5′- GCGACTGTAAACATCCTCGACTGG-3′.

MI30A-5P R: 5′-GCAGCTGCAAACATCCGAC-3′

NoTCH1-F: 5′-GGACGTCCATCTGGCTCAG-3′.

NoTCH1-R: 5′-ACATCTCGGACGCACTGG-3.

GAPDH-F: 5′-TCCGTGGTCCACGAGAACT-3

GAPDH-R: 5′-GAAGCATTTGCGGTGGACGAT-3′

### Recombinant adenovirus transfection

BMSCs were seeded in 6-well plates for 24 h. After removal of the growth medium, the fresh medium without serum and penicillin–streptomycin was added to the cells. The recombinant adenovirus vector (Invitrogen) of HCG18 and NOTCH1 or the control was mixed with Lipofectamine2000 (Sigma) in EP tube and added to the cells, respectively. The expression vector was fully mixed with the medium and continued to culture for 48 h. Western blot and qRT-PCR were used to detect the expression of the target genes. All recombinant adenovirus were designed and synthesized by Invitrogen.

### Oligonucleotide transfection

Oligonucleotide sequences and controls including the miR-30a-5p mimetic were synthesized by GenePharma (Shanghai, China). 50 nM oligonucleotides were transfected into cells using Lipofectamine 2000 (Invitrogen, Carlsbad, CA).

### ALP staining and Alizarin red S staining

Cells were seeded in six-well plates and cultured in osteogenic differentiation induction medium for 12 d. Alkaline phosphatase (ALP) staining was conducted using a nitro-blue tetrazolium chloride (NBT)/5-bromo-4-chloro-3-indolylphosphate toluidine (BCIP) staining kit (CoWin Biotech, Beijing, China) after fixation in 95% ethanol at room temperature. ALP activity was measured using an ALP assay kit (Nanjing Jiancheng Bioengineering Institute, Nanjing, China) following the manufacturer’s instructions and normalized to the total protein contents as determined using the BCA method (Thermo Fisher Scientific, Rockford, IL, USA). 2% Alizarin Red S (ARS; 2%; Sigma-Aldrich; Merck KGaA, Darmstadt, Germany) was used to detect matrix mineralization using an inverted microscope (Olympus IX73; Olympus Corporation, Tokyo, Japan). Individual experiments were repeated at least three times.

### Luciferase assay

A wild-type HCG18 (HCG18 wt) reporter plasmid or a mutated reporter plasmid (HCG18 mut) was constructed using the pmirGLO luciferase vector (Promega, Madison, WI). The luciferase reporter plasmid was transfected into HEK-293 T cells. The miR-30a-5p mimic or negative control was then transfected with Lipofectamine 2000 (Invitrogen). Luciferase activity was then measured by a dual luciferase assay system (Promega).

### RNA pull down assay

The biotinylated negative control, miR-30a-5p, miR-30a-5p-Mut (RiboBio) was transfected into BMSC for 48 h. Whole cell extracts were then incubated with M-280 streptavidin magnetic beads (Invitrogen, USA) at 4 °C for 4 h. The coprecipitated RNA was then isolated by lysis buffer containing proteinase K (Invitrogen) and 10% SDS and detected by qRT-PCR analysis. The sequence of negative control was 5′-Bio-ACGCATGCGAGTTCACGATCAGGTC-3′; The sequence of miR-30a-5p probe was 5′-Bio-UGGCCAGGCCUACAAGCUCAUG-3′; The sequence of miR-30a-5p-Mut probe was 5′-Bio-UUUGGUCCCCUUCAACCAGCUG-3′.

### ALP activity measurement

The transfected hBMSCs cells were harvested, the medium was removed, and ALP activity was measured by the ALP Colorimetric Assay Kit (Donglian, Jiangsu, China).

### Western blot analysis

Total proteins were extracted, and protein concentrations were quantified using the BCA Protein Assay Kit. The membrane was blocked and incubated with rabbit anti-Runx2 (1:1000, ab192256, Abcam), anti-OCN (1:1000, ab13420, Abcam), anti-OPN (1:1000, ab75285, Abcam) primary antibody, anti-NOTCH1 (1: 1000, ab8925, Abcam) and anti-GAPDH (1:10,000, ab181602, Abcam) at 4 °C overnight. Then a horseradish peroxidase-conjugated secondary antibody was added. Western blot analysis was performed according to the references (Welinder and Ekblad 2011). The western blot experiments were performed in three replicates.

### Construction of lentiviral vectors for shRNA HCG18

The recombinant HCG18-RNAi lentivirus and negative control lentivirus were obtained from Wanlei Biological Technology Co., Ltd. (Shenyang, China). Twenty mice were randomly divided into four groups by random scale, as follows: (1) Con, (2) HU, (3) HU + shNC, and (4) HU + shHCG18 (N = 5). The HU mice were hung from the top of the cage by the tail at a 30°angle with only the forelimbs touching the floor, which allowed them to move and access food and water freely. Before hindlimb unloading, mice in the experimental groups (HU + shNC and HU + shHCG18) were injected with 2 mg/kg plasmids every day for 3 consecutive days. After 3 weeks of tail suspension, all mice were in normal condition without abnormal death. Mice were euthanized, and the bilateral femurs and tibiae were harvested. In animal experiments, the researchers who took samples and tested the indicators did not know the grouping situation.

### microCT analysis

Each mouse femur was fixed in 4% paraformaldehyde for 24 h and scanned by a microCT scanner (Siemens, Germany) with energy of 80 kV and 500 mA. Femurs were scanned over a total angle of 360° at incremental angles of 0.5°. The scanning time was 800 ms/frame at a resolution of 10.44 μm. The region of interest (ROI) represents the microstructure of the femur, which was 1500 μm above the proximal epiphyseal growth plate and was selected as a 2.5 × 2.5 × 3 mm^3^ cube. The parameters, including BMD, BV/TV, Tb.Th, Tb.N, BS/BV, Tb.Sp and TbPF were analyzed by COBRA software for microCT. These data were collected for blinded analyses.

### Histology

Harvested femurs were fixed in 4% paraformaldehyde, decalcified in 10% ethylenediaminetetraacetic acid (Beyotime Biotechnology, Shanghai, China), and embedded in paraffin. For histological analysis, bone sections were stained with H&E according to the manufacturer’s protocol (Sigma, USA).

### Statistical methods

The monitoring data were analyzed by SPSS19.0 statistical software. The results of data analysis were showed as mean ± standard deviation (SD). Multigroup data analysis was analyzed by one-way ANOVA. LSD (Least-Significant Difference) test was used for subsequent analysis. *P* < 0.05 meant the difference was significant.

## Data Availability

The datasets used and/or analyzed during the current study are available from the corresponding author on reasonable request.

## Abbreviations

BMSCs: Bone marrow mesenchymal stem cells
OP: Osteoporosis
mean ± SD: Mean ± standard deviation
PTH: Parathyroid hormone
LncRNA: Long non-coding RNA
HU: Hindlimb unloaded
mESCs: Mouse embryonic stem cells
